# Climatic backdrop for Pueblo cultural development in the southwestern United States

**DOI:** 10.1038/s41598-022-12220-6

**Published:** 2022-05-24

**Authors:** Victor J. Polyak, Yemane Asmerom, Matthew S. Lachniet

**Affiliations:** 1 Earth and Planetary Sciences, University of New Mexico, Albuquerque, NM 87109 USA; 2 Department of Geoscience, University of Nevada, Las Vegas, Las Vegas, NV 89154 USA

**Keywords:** Climate sciences, Environmental sciences

## Abstract

While climatic triggers for collapse and population migrations of ancestral Pueblo communities have been proposed, little is known about the overall climatic backdrop for the entire pre-Hispanic Pueblo period (ca. 1300 to 460 B2K). Here, we report data from stalagmite HC-1, from Hidden Cave, Guadalupe Mountains, New Mexico, covering the past 3400 years, showing an interval of increased frequency of droughts from 1260 to 370 yr B2K that is coeval with the entire pre-Hispanic Pueblo period. Our record suggests that this puebloan Late Holocene climatic interval was the most arid and highly variable climatic period of the last 3400 years. Climatic conditions favoring the introduction of cultivation existed prior to the Pueblo period during more pluvial-like conditions from at least 3400 to 1260 yr B2K. Hence, the change from the Desert Archaic/Basketmaker to Pueblo cultures was associated with a quick transition to increasing aridity into and through the Pueblo period associated with greater urbanization and the establishment of pueblo population centers.

## Introduction

The link between climate and culture in the southwestern United States (SW USA) has been discussed extensively in the literature ^1–5^, although the overall Late Holocene climatic backdrop is not well known prior to 1200 years ago, which is beyond most compelling dendroclimatic records^6,7^. For example, there is a need to place into context Late Holocene megadroughts over several millennia. The broadly accepted view is that megadroughts lead to cultural disintegration of individual pueblo sites during the pre-Hispanic Pueblo period^1^. Even the causal relationship between drought and dramatic cultural shifts has been challenged^8^. A clear understanding of the climatic backdrop of the Pueblo period compared to the entire cultural and climate history for the last several thousand years is essential to comprehend the role of climate on culture during the Pueblo period. This perspective is needed to identify and interpret the numerous megadroughts to have reportedly affected individual pueblos. The SW USA cultural history during the last 4000 years, the Late Holocene climatic epoch, is categorized as Basketmaker (pre-pueblo) and the cultural stages of the Pueblo period using the Pecos classification offered nearly 100 years ago^9^. That classification is still broadly used today^1,2^. Climatically, the Altithermal and Medithermal reported by Antevs^4^ in the early half of the twentieth century were based on an assessment of climate from geologic evidence in the Great Basin and SW USA that is roughly coeval with the Middle and Late Holocene climates, where the Altithermal (the Middle Holocene) was considered an interval of drought conditions from ~ 7500 to ~ 4000 yr B2K in the SW USA referred to as the Long Drought. This compares remarkably well with pronounced extensive aridity reported between 9000 and 4500 yr B2K based on a synthesis of SW USA paleoclimate records^10^. The Medithermal (the Late Holocene) was considered cooler and had more fluctuations of greater moisture, an assessment that is also still held^3,10–12^. The broader scaled culture-climate association is complicated by the lack of coherency between records, which may in part be due to the lack of records with comparable high-resolution chronologies.

**Figure 1:**
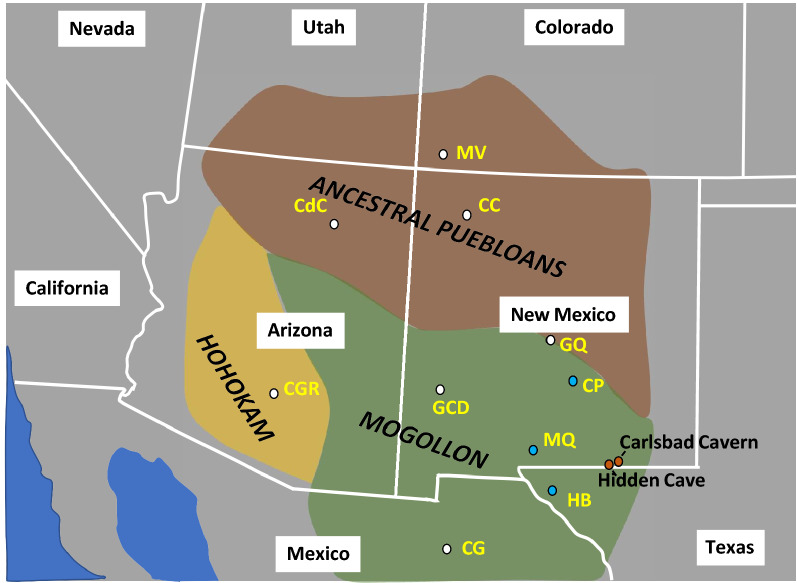
The Hohokam, Anasazi, and Mogollon cultures represent generalized ancestral pueblo cultures of southwestern North America as commonly depicted in the literature^14–16^. Hidden Cave and Carlsbad Cavern are in the eastern Mogollon region. Major pueblo complexes of these regions are Casa Grande Ruins (CGR) and Canyon de Chelly (CdC) in Arizona, Casas Grande (CG) in northern Mexico, Mesa Verde (MV) in Colorado, and Chaco Canyon (CC), Grand Quivera (GQ), and Gila Cliff Dwellings (GCD) in New Mexico. Smaller pueblo sites closest to Hidden Cave are pueblos in the Hueco Bolson (HB) in west Texas, and Madera Quemada pueblo (MQ), and pueblos near the Capitan Mountains (CP) in southern central New Mexico. Google Earth was used to construct state borders (http://www.google.com/earth/index.html).

Long, continuous, high-resolution paleoclimate studies such as those from stalagmites are helpful as climatic backdrops to American cultural changes^3,13^, and they extend beyond tree-ring paleoclimate records that are typically applied to the study of pueblo cultures. Here we offer a new record covering much of the Late Holocene from stalagmite HC-1 collected from Hidden Cave in the Guadalupe Mountains of southeastern New Mexico (Supplementary Information S1 and Fig. S1). This stalagmite yields a remarkable record of growth from ~ 3400 to ~ 50 yr B2K that is offered as a template for Late Holocene climate change for the eastern portions of the SW USA that provides the climatic context for the cultural changes that took place in the region (Fig. 1). The Guadalupe Mountains region is important because it is located near areas suitable for rain-fed maize agriculture, which in other areas of the SW USA sustained pueblo populations for extended time intervals^17^. Further, caves from the Guadalupe Mountains region are sensitive to summer infiltration due to a strong summer rainfall peak. As such, speleothem paleoclimate records from this area should be sensitive to changing summer and winter precipitation amounts, and in particular, to changes in hydroclimate associated with drought cycles.

**Figure 2:**
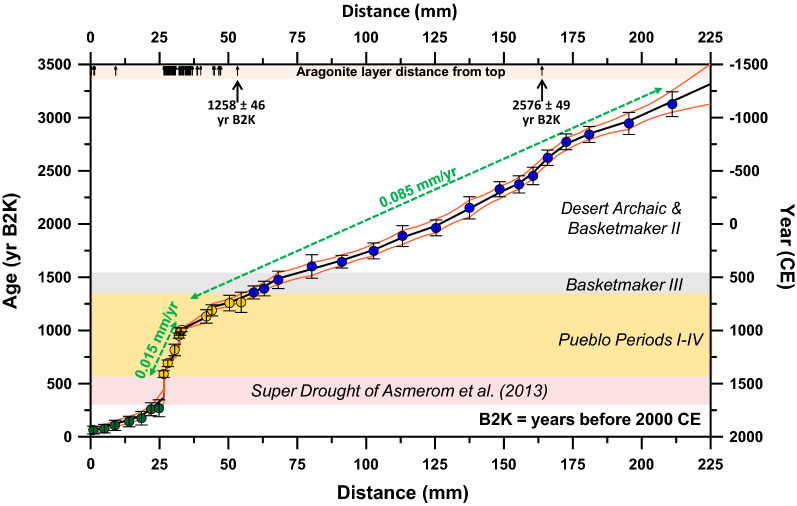
Stalagmite HC-1 high-resolution chronology consists of 34 uranium-series dates. Three intervals of growth are apparent, and compared with cultural periods, the most obvious association is the interval of slow stalagmite growth that takes place coincident with the Pueblo cultural stages. The age model 2σ uncertainty envelope shown as orange curves was generated using COPRA^23^. The symbols for aragonite layers (small black arrows) are only tied to distance (x-axis).

The most influential proxy data from stalagmite HC-1 are the changes in mineral assemblages between calcite and aragonite. We support these data with stable isotope time-series, which exhibit evidence of kinetic fractionation that hinders it’s use as an isotopic equilibrium tool. Change from calcite to aragonite indicates a transition from wet to arid conditions^18^, although prior calcite/aragonite precipitation can complicate this particular interpretation. Prior calcite/aragonite precipitation as stalactites or in epikarst above a cave would be expected in caves with large entrances in semiarid areas and would favor a change from calcite to aragonite in stalagmites during a transition from wet to dry climate. While this would favor a calcite-to-aragonite transition equating to wet-to-dry climate, another issue related to prior calcite/aragonite precipitation is that it could drive long-term (1000 s of years) progressive changes in stalagmite proxy trends that are not climate change related^19^. Shorter-term (> 30 to 100 s of years) variability and the reverse of such progressive trends, however, should reflect climate change. We leverage the growth and mineral assemblage in stalagmite HC-1 and explore the potential of stable isotope disequilibrium relationships to provide a climate record that complements an earlier growth record for the region^3^. Stalagmite HC-1 has a high sensitivity to climate change and a robust chronology (Supplementary Table S1; Fig. 2) from advances in U-series methods^20,21^.

## Results and discussion

### Stalagmite growth, mineral assemblage, and U-series chronology

To document changing stalagmite growth and mineral assemblage, we analyzed thin sections from the central growth axis of stalagmite HC-1 to construct a high-resolution time-series of alternating calcite and aragonite from grayscale imaging, in which the aragonite layers are darker in transmitted light (Supplementary Information S2; Fig. S2). We interpret the aragonite layers to represent episodes of drier climate, an idea supported by the slower growth of aragonite and associated growth hiatuses, making them physically distinct climate indicators, and that these calcite and aragonite layers represent original growth that is interrupted by hiatuses and inclusion levels that can record changes in environmental conditions^22^. Stalagmite HC-1 hosts several aragonite layers tied to a high-resolution chronology (Fig. 2). Our interpretation is supported by previous work that has demonstrated a change to aragonite from calcite is associated with increased aridity in arid and semi-arid regions, resulting from increased drip water supersaturation with aragonite associated with slower drip intervals and lower cave humidity in moisture-limited regions like the SW USA (Supplementary S3; Fig. S3).

**Figure 3:**
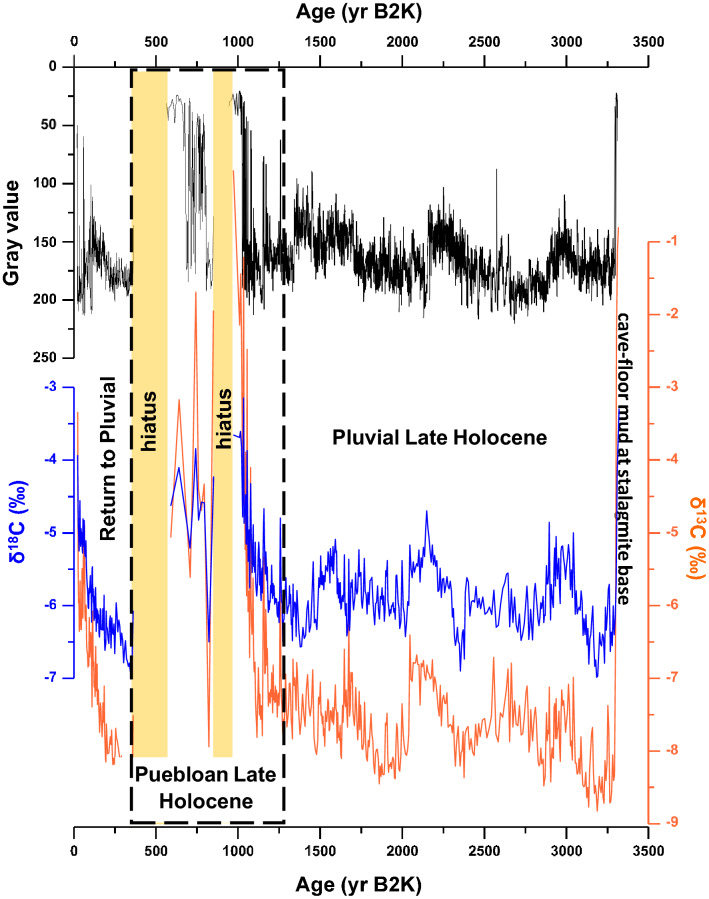
Stalagmite HC-1 grayscale, and stable isotope COPRA-generated time-series showing disruptions to each proxy coeval with the Pueblo period. Two significant growth hiatuses (or intervals of very slow growth) also occur during the Pueblo period.

Stalagmite HC-1 chronology uses 34 U-series ages (Supplementary Table S1; Fig. 2) to construct the δ^13^C, δ^18^O, and gray value time-series (Supplementary Table S2 and Fig. 3) using COPRA^23^. Growth of the stalagmite began 3320 ± 200 years before 2000 CE (yr B2K, extrapolated basal age). The thickest sequence of uninterrupted stalagmite growth is a 1300-year interval from 2576 ± 49 yr B2K to 1258 ± 46 yr B2K, where no distinct aragonite layers/hiatuses formed (Supplementary Fig. S4). The average stalagmite growth rate from ~ 3320 to ~ 1260 yr B2K is 0.085 mm/yr (Fig. 2). Markedly slower and interrupted growth occurred between 1258 and 366 ± 80 yr B2K, an interval with an average growth rate of 0.015 mm/yr that includes two 1–2 mm-thick aragonite layers, and several other hiatuses and thinner aragonite layers. There is however, a very noticeable change in grayscale (fluid inclusion density) at 1416 ± 50 yr B2K. The two thick aragonite layers are each associated with a growth hiatus from ~ 950 to ~ 860 yr B2K and ~ 560 to ~ 370 yr B2K. About 250 years of fast and continuous calcite growth followed the long ~ 210-year growth hiatus interpreted as an extended period of megadrought conditions dubbed a ‘super drought’^24^. Note that comparing our record with individual events such as the early twentieth century droughts or tree-ring defined megadroughts is made difficult by our larger 2σ absolute age uncertainties that range from ± 15 to ± 80 years from 3100 to 20 yr B2K. These uncertainties, however, accurately define our sub-periods. Also, we consider the change from calcite to aragonite growth or growth hiatuses in stalagmite HC-1 as subtle but substantial changes in climate, rather than extreme, that exceed a threshold related to this stalagmite’s growth response to drip water chemistry and cave enviornment. For this stalagmite record, we consider a drought to be a prolonged period of effectively dry weather that translates to aragonite growth and growth hiatuses. Pluvial is considered to be a prolonged normal or wet period that translates to continuous calcite growth.

### Covariation of δ^18^O versus δ^13^C values as a climate proxy

**Figure 4:**
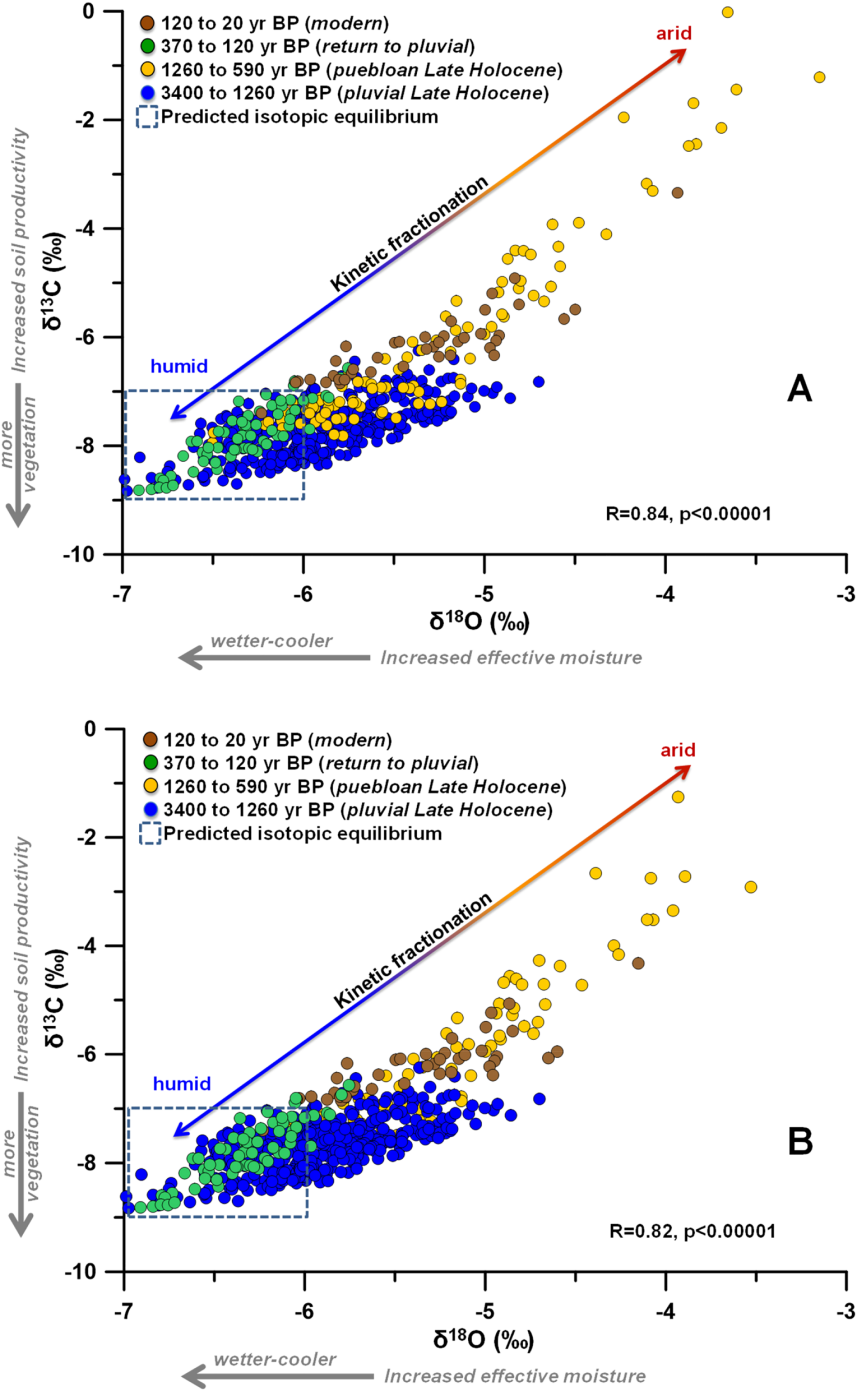
δ^18^O versus δ^13^C values illustrate an isotope fractionation trend due to evaporation and rapid CO_2_ degassing. This trend is color-coded for each climatic sub-period and exhibits higher values along the trend that are coincident with the Pueblo period and with the last 150 years. Top and bottom graphs represent the same data uncorrected (**A**) and corrected (**B**) for percent of aragonite, respectively (Supplementary Information S4).

We pair the mineral assemblage observations to the absolute values of stable isotopes and the degree of δ^18^O/δ^13^C covariation. The correlation of δ^18^O and δ^13^C in stalagmite HC-1 is strong (R = 0.82, *p* < 0.00001). We interpret the covariation and strong Pearson correlation as driven by kinetic fractionation with higher values representing greater epikarst or drip water evaporation associated with aridity. The highest δ^18^O and δ^13^C values are associated with aragonite layers. Both δ^18^O and δ^13^C values begin to increase from baseline values of around -6.0 and -8.0‰, respectively, for about a century prior to the occurrence of the aragonite layers (Fig. 3). The aragonite layers have δ^18^O and δ^13^C values around -4 and -2‰ VPDB, respectively. We also observe that intervals of stronger correlation (higher Pearson R values) are associated with a greater spread in absolute δ^18^O and δ^13^C values. We thus focus on the climatic sub-periods broadly defined by stalagmite HC-1 mineral assemblage that show δ^18^O and δ^13^C covariability (Fig. 4; Supplementary Information S4). Although the precise cause of this covariation may be multivariate, we favor an interpretation that higher δ^18^O and δ^13^C values indicate a drier, more evaporative climate. In contrast, lower values indicate near-equilibrium precipitation of calcite during more humid climate. For example, covariation between δ^18^O and δ^13^C values has been reported as a proxy for evaporation/aridity in lake^25,26^ and speleothem records^27,28^, and due to prior calcite precipitation tied to aridity in a Great Basin stalagmite record^29^. Support for our interpretation is the increase in stable isotope values preceding periods of aragonite precipitation, and the higher absolute δ^18^O and δ^13^C values of the aragonite. In contrast, the interval of continuous calcite growth from 3320 to 1260 yr B2K is characterized by calcite with lower δ^18^O and δ^13^C values, which we interpret to be close to isotopic equilibrium with the drip waters and supports an interpretation of pluvial-like climate during this time (Fig. 4). For example, the δ^18^O values of drip waters from > 10 sites in Carlsbad Cavern, also in the Guadalupe Mountains, have a mean value of -7.5 ± 0.2‰ VSMOW^30,31^. At a cave temperature of ~ 11.0˚C at Hidden Cave, the expected δ^18^O values of calcite and aragonite precipitated at or near equilibrium are -6.5 and -6.0‰ VPDB, respectively, using the fractionation equations for abiogenic calcite^32^ and biogenic aragonite^33^ as reformulated in Lachniet^34^. An abiogenic value for aragonite using the Kim et al.^35^ equation is -6.6 ‰ VPDB. In comparison, the δ^18^O values of calcite deposited in the pre-Pueblo sub-period are -5 to -7‰ VPDB, which fall within the range of those expected for equilibrium calcite precipitation and support our interpretation that the pre- and post–pre-Hispanic Pueblo climatic sub-periods were less arid (Figs. 3 and 4).

Increased evaporative conditions would lead to decreasing relative humidity in the cave air, slower drip rates and possibly enhanced stalactitic prior calcite/aragonite precipitation resulting in growth hiatuses and aragonite layers. The stalagmite interval representing the slowest growth, between 1260 and 370 yr B2K, contains multiple aragonite layers, two growth hiatuses, and the highest δ^18^O and δ^13^C values, all of which supports an interpretation of greater aridity. The interval from 370 to ~ 120 yr B2K was a time of near-equilibrium continuous calcite growth and supports an interpretation of a return to a wetter climate. From ~ 120 to ~ 50 yr B2K, multiple aragonite layers and higher values of δ^18^O and δ^13^C in stalagmitic calcite and aragonite represent an increasingly drier climate into modern times. Covariation of δ^18^O versus δ^13^C values appears to reveal humid and arid stalagmite growth intervals suggesting that this approach represents a viable climate proxy in stalagmites from evaporative cave sites.

### Transition from middle to Late Holocene

The interval from the end of the Middle Holocene in our study area at 4250 yr B2K^36^ to the beginning of more pluvial conditions starting ~ 3400 yr B2K is less well-represented by our stalagmite collection and studies, which suggests that this is a subtle climatic transition in the SW USA. The beginning of the Late Holocene climate transition to wetter and cooler conditions at 4250 yr B2K in the Great Basin and SW USA^10^ coincides with the timing of the well-documented introduction of maize into the SW USA^37^. In Grand Canyon, this is the time exactly represented by split twig figurines, animal figures constructed of single elongated split twigs made between 4400 and 3700 yr B2K, that are reported as linked to an increase in climate variability in that area possibly driven by the onset of the increased influence of the El Niño/Southern Oscillation^38^. Late Archaic and early Basketmaker cultures (**S5**) implemented the cultivation of maize coincident with this earliest Late Holocene interval^37^ that we interpret to be an onset of slightly wetter climate in the SW USA^3^, but slightly drier than the following two millennia starting ~ 3400 yr B2K. Examination of both climate and human responses necessitates climatic sub-periods defined by stalagmite HC-1 that also satisfies the timing of cultural periods; for instance, the wettest sub-period, herein referred to as the pluvial Late Holocene, and the driest sub-period, herein referred to as the puebloan Late Holocene, fit well with the defined cultural periods^2^.

### Pluvial Late Holocene

Our study of stalagmite HC-1 indicates faster and more uniform continuous calcite growth during an interval starting ~ 3400 yr B2K and ending ~ 1260 yr B2K, an interval during which stalagmite HC-1 exhibits only two distinct brief growth hiatuses defined by thin aragonite layers at ~ 2576 ± 87 yr B2K and 1258 ± 62 yr B2K (Supplementary Fig. S4). The HC-1 stable isotope time-series for this interval compares well with a Belizean and a Spanish stalagmite time-series, with all three records potentially tied to northern hemisphere temperature and climatic behavior (Supplementary Information S6; Fig. S5). The interval between these two brief hiatuses is coeval with an interpreted wettest Late Holocene interval from a study of spring mounds in western New Mexico^11^. Increased effective moisture in the SW USA at this time coincides with canal and early agricultural development on the New Mexico Colorado Plateau 3000 to 1000 years ago^39^. We consider this interval from 3400 to 1260 yr B2K to be the effectively wettest and likely coolest climatic phase since the middle Early Holocene in our study area^3,40,41^. This pluvial Late Holocene climatic interval defined by stalagmite HC-1 growth is remarkably coeval with the Basketmaker II and Basketmaker III cultures of the SW USA. Our stalagmite HC-1 record cannot discern Basketmaker III climate from Basketmaker II (Supplementary Information S5). By our interpretation, this pluvial sub-period from ~ 3400 to ~ 1260 yr B2K suggested by our stalagmite growth would have been a good time for populating the SW USA, particularly if the wetness were associated with summer monsoon rains that fed maize agriculture^2,17^.

### Puebloan Late Holocene

**Figure 5:**
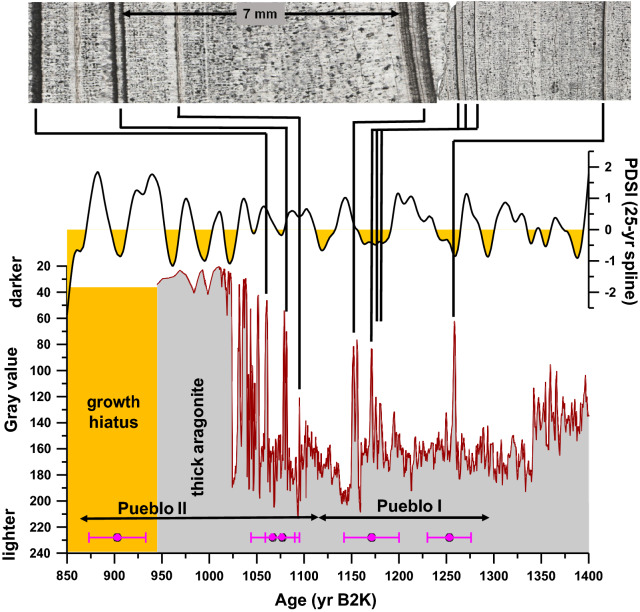
Gray time-series and thin section images through Pueblo I. (**A**) The thin section image shows the first cluster of aragonite (dark) layers after 1300 yr B2K illustrating the onset of drier conditions going into the Pueblo period. Several decades later, five aragonite layers (droughts) occur within a span of ~ 30 to 40 years (~ ≤ 8 years per drought defined by aragonite layers) starting at 1180 ± 125 yr B2K. The four dates in magenta between 1050 and 1300 yr B2K represent the timing of droughts from charcoal layers in ice deposits in Cave 29 of El Malpais National Monument^42^. The date at 913 yr B2K on charcoal attached to a corrugated pottery sherd also likely represents a drought^42^. All of these are comparable with droughts represented by aragonite layers in stalagmite HC-1. PDSI (Palmer Drought Severity Index) for much of the greater Southwest for the area defined as 29.71°N—38.83°N and 111.64°W—101.64°W is plotted as a 25-yr spline^43^.

**Figure 6:**
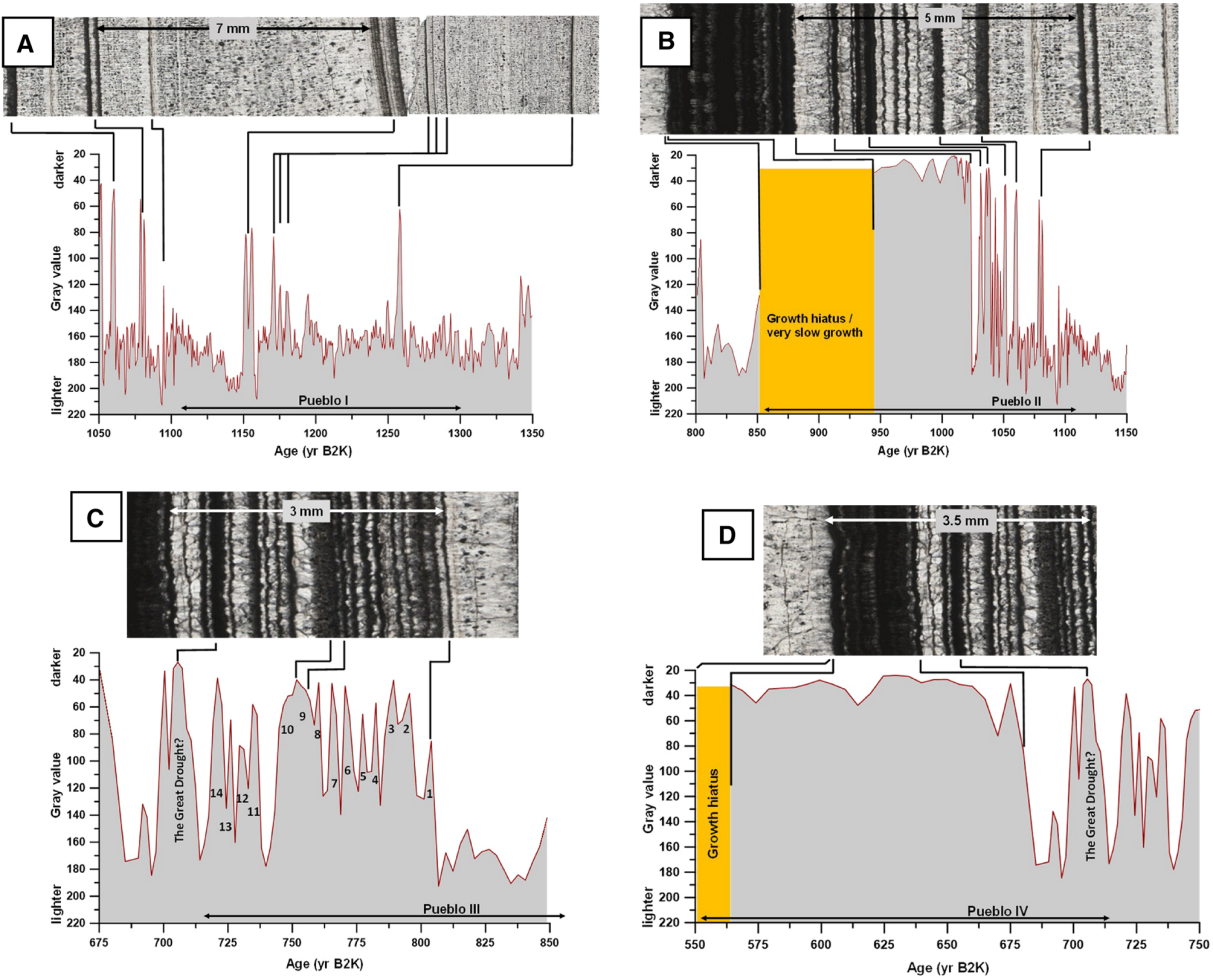
Gray time-series and thin section images through Pueblo cultural stages. (**A**) The thin section image shows the first cluster of aragonite (dark) layers after 1300 yr B2K illustrating the onset of drier conditions going into the Pueblo period. (**B**) Pueblo II is defined by closely spaced aragonite layers and ends with a thick aragonite layer and terminal growth hiatus. (**C**) Thin section image shows Pueblo III starting as calcite growth and transitioning to numerous more evenly spaced aragonite layers. (**D**) Pueblo IV consists of mostly thick aragonite layers and the period ends in a lengthy growth hiatus.

A cluster of aragonite layers and growth hiatuses are exhibited in stalagmite HC-1 during this interval from 1260 to 370 yr B2K, a climatic period that is remarkably coeval with the Pueblo cultural stages I-IV (Figs. 2, 3 & S6; Supplementary Information S5), and thus referred to as the puebloan Late Holocene. The abundant aragonite layers and brief hiatuses in stalagmite HC-1 indicate that stalagmite HC-1 growth was disrupted during this interval due to increased aridity and climate variability (Supplementary Fig. S4). Pueblo I starts at 1300 yr B2K^2^, which is close to the first aragonite-defined drought at 1258 ± 62 yr B2K marking the end of two millennia of pluvial climate. Pueblo I ends ~ 1110 yr B2K just prior to another set of droughts, but between these sets of droughts at the beginning and end of this period is ~ 50 years of average calcite growth indicating that this interval bounded by droughts was pluvial-like climate in the middle. Aragonite-defined droughts were more numerous during the 190-year Pueblo I stage than during all previous decades back to 3400 yr B2K. But our stalagmite-based interpretation suggests that Pueblo I experienced far fewer of these aragonite-defined droughts than the next three Pueblo stages as shown in Fig. 5, a gray time-series that shows an increase in aragonite layers from Pueblo I to Pueblo II, which is compared to a tree ring record from the living blended drought atlas^43^ for our study area. A comparison of Pueblo I-IV grayscale histograms is offered in Fig. 6.

Pueblo II in the stalagmite HC-1 record is a 255-year interval of more frequent aragonite-defined droughts (Fig. 6). The age model placed an ~ 90-year growth hiatus or very slow growth after 950 yr B2K, indicating that the SW USA experienced relief from these droughts around 860 yr B2K. Pueblo II would appear to have been much drier than Pueblo I**. ** Pueblo III in the stalagmite HC-1 record is an ~ 140-year span of numerous but less severe droughts compared to Pueblo II. Our HC-1 record suggests that Pueblo II was a period of highly variable climate with ~ 14 evenly spaced aragonite-defined growth hiatuses (droughts; Fig. 6C). This evenly spaced climate variability indicated by the occurrence of aragonite layers in stalagmite HC-1 from the late Pueblo II into early Pueblo III, by our interpretation, would have been an ~ 140-year interval of ‘predictable’ climate variability, and possibly easier for cultures to adapt.

From 685 to 560 yr B2K, aragonite layers suggest that the driest interval of our puebloan Late Holocene record was Pueblo IV, which is represented by slower stalagmite growth, aragonite layers, and the lengthiest growth hiatus in our 3400-year record (Fig. 6D). The end of the Pueblo IV stage and the beginning of the modern period is marked by a significant ~ 210-year growth hiatus in stalagmite HC-1 from 560 ± 30 to 370 ± 41 yr B2K, the Super Drought^24^. The Spanish explorers made their way into the SW USA 460 yr B2K during the stalagmite-indicated Super Drought, when ancestral American populations, clustered along permanent water ways^44^, were by this time well-adapted to drought.

### Return to pluvial climate

Continuous calcite growth resumed at 370 ± 41 yr B2K for ~ 250 years in stalagmite HC-1 and at its initiation had low δ^18^O and δ^13^C values. From about 370 to 125 ± 30 yr B2K, climatic conditions were pluvial-like, which seems consistent with tree-ring records^6,7^. However, after 125 yr B2K, droughts became more frequent, and δ^18^O and δ^13^C values gradually increased to the top of the stalagmite. The aragonite layer at the stalagmite top shows that this stalagmite was broken by cave visitors during an interval of drought at 60 ± 40 yr B2K.

### Comparison to more regional cultural and climatic changes

We emphasize the aridity and climate variability of the puebloan Late Holocene sub-period (1260 to 370 yr B2K). This seems to be a climatic interval that affected other distant cultures as well, such as the hunters and gatherers along the coast of California, the Mississippian mound-builders, and Mesoamericans. The puebloan Late Holocene sub-period is roughly coeval with cool sea surface temperatures along the California coast that caused cool dry climatic conditions on land from ~ 1550 to 700 yr B2K that was described as a time of increased sociopolitical complexity^45^. Settlements along the more southern Mississippi Valley may have experienced similar climatic and cultural changes^46^, where drought conditions may have driven people from southern drought-stricken areas to more northern less-drought-stricken areas by 800 yr B2K^47^. Simultaneously, the Mesoamerican region experienced more complex climatic variability between Maya highlands and lowlands linked to the rises and falls of several Mayan societies^48–51^.

Most SW USA climate and culture studies focus on the climatic comparison between individual pueblos or specific droughts related to the migration, disintegration and abandonment of pueblo societies^1,2^. A study of seven SW USA cultural traditions from 1100 to 500 yr B2K could not show a statistically robust relationship between drought and dramatic cultural change^8^, which is in contrast to many earlier studies. Our study differs because it contextualizes the entire pre-Hispanic Pueblo period as the climatically driest part of the Late Holocene. Our stalagmite HC-1 results clearly suggest that the pluvial Late Holocene and the post-Pueblo I-IV pluvial sub-periods that bookended the interval defined by Pueblo I-IV were effectively wetter. This would argue for a possible interpretation that Ancestral American cultures in the SW USA shifted to pueblo development to adjust to ever-increasing aridity, forcing pre-Pueblo settlements towards greater integration and urbanization as a coping strategy. This interpretation contrasts with the development of three Mayan cities (Teotihuacan, Tula, and Tenochtitlan; Mexico) during transitions from drier to wetter climate^52^, but both scenarios seem to demonstrate human adaptation to climate change. Such adaptations are proposed by Bocinsky and Kohler^17^ where more extreme environmental changes will force human behavioral changes that drive adoption or development of alternative strategies.

## Conclusion

Drought has been blamed for the abandonment of pueblos and episodic human migration during the North American Pueblo period. Much interest in this subject has been focused on individual megadroughts and pueblo sites. The rise of the pre-Hispanic Pueblo period and its climatic backdrop remain enigmatic. Our continuous high-resolution climate record for the SW USA from stalagmite HC-1 serves as a climatic backdrop to the cultural periods including the interval that extends through pueblo cultural development. The stalagmite record shows that climate was effectively wetter during Basketmaker II and III, and drier and more variable from Pueblo I-IV. We suggest that the onset of a drier climate was a factor that forced ancestral Americans to develop alternative strategies to subsist, leading to greater urbanization and explaining the development of pueblo communities. Our view is that pueblo cultural development was a way of coping with overall drier and more variable climatic conditions that extended through the entire pre-Hispanic Pueblo period.

## Methods

Uranium-series and stable isotope analyses methods typical of this type of study are well represented in the literature^21,24^. Subsample powders (20–200 mg) were dissolved in 15 N HNO_3_, mixed with a mixed ^229^Th – ^233^U – ^236^U spike solution. One to two drops of perchloric acid (0.07 ml) were added to dissolve organics and help equilibrate the sample and spike. Once dried down, the spiked subsample solution was re-dissolved in 7 N HNO_3_. Anion resin in columns is cleaned, then conditioned with 7 N HNO_3_. Thorium was separated in the columns using 6 N HCL, and uranium was collected using water. U and Th were analyzed on a Thermo Neptune multicollector inductively coupled plasma mass spectrometer using a static measuring routine where all isotope signals are collected in Faraday cups except for ^230^Th and ^234^U, which were collected in a secondary electron multiplier. Gain values between the cups and multiplier were monitored during the runs using an in-house ^230^Th – ^229^Th solution and the NBL-112 U standard.

Most speleothems analyzed in the Guadalupe Mountains have high initial ^230^Th/^232^Th values making age corrections more challenging. These values negatively correlate non-linearly with ^232^Th concentration, where higher ^230^Th/^232^Th values are probably derived as ^230^Th contribution from the bedrock at the time of stalagmitic calcite/aragonite crystallization, and where lower ^230^Th/^232^Th values are probably derived as ^230^Th contribution from the soil^53,54^. This relationship is probably related to differences in residence time in the bedrock and soil thickness. The initial ^230^Th/^232^Th correction to the uranium-series ages is substantial compared to the commonly used 4.4 × 10^–6^ atomic ratio. See Supplementary Table S1. Consequently, larger errors accompanying these values result in larger absolute uncertainties on the ages. A better understanding of these corrections^53^ and improvements in uranium-series methods^21^ have increased our accuracy and reduced these uncertainties to half in the more recently measured ages that make up our new HC-1 chronology. The uncertainties related to milling trench diameter and ability to follow along layers is ~ 5–15 years and smaller than the final analytical uncertainties.

Thin sections were custom-cut to ~ 50 rather than the standard 30 µm thickness to enhance the imaging of stalagmite layering (annual and decadal banding and hiatuses). Thin section images were collected using a Nikon Coolscan V slide scanner to minimize distortion. Images were also collected on a Hirox KH-7700 digital microscope. Grayscale histograms were created using Digital Micrograph version 1.71.38. Grayscale was collected from transmitted light through thin sections at 50 to 100-pixel transect widths (~ 1 mm), which more accurately depicts the growth history and helped filter out some of the noise. The grayscale time-series was constructed by tying the grayscale histogram to our uranium-series chronology (Fig. 2). Micro-cracks along the layering in the stalagmites produce false layers in the grayscale time-series and were manually removed from the collected data for the final dataset.

Stable isotope powders were milled at 0.5 mm resolution, except for two areas milled at 0.25 mm resolution. Powders for δ^13^C and δ^18^O analysis were analyzed at the Las Vegas Isotope Science Lab (LVIS) of the University of Nevada, Las Vegas. Stalagmite powders were reacted with three drops (0.1 ml) of anhydrous 104% phosphoric acid at 70 °C in a Thermo Electron Kiel‐IV automated carbonate preparation device coupled to a Delta V Plus mass spectrometer. Values are reported in standard permil (‰) notation with respect to Vienna Pee Dee Belemnite (VPDB) with precisions of 0.08 and 0.06‰ VPDB for δ^18^O and δ^13^C, respectively, relative to an internal standard USC-1, which was run 42 times during the 553 sample analyses. δ^13^C, δ^18^O, and gray value time-series were constructed using COPRA^23^.

## Supplementary Information

Supplementary Information.

Supplementary Tables.

## Data Availability

All data needed to support the conclusions of this paper are included in the main text and supplementary files.
