# Supplementary material for: Climatic backdrop for Pueblo cultural development in the southwestern United States
Source: Sci Rep. 2022 May 24;12:8723. doi: 10.1038/s41598-022-12220-6 (PMC9130131; doi:10.1038/s41598-022-12220-6)
Supplement: Supplementary file 1 — Supplementary Information. [file 41598_2022_12220_MOESM1_ESM.pdf]

Climatic Backdrop for Pueblo Cultural Development in the Southwestern United States

Victor J. Polyak[1*], Yemane Asmerom[1], Matthew S. Lachniet[2],

[1] Earth & Planetary Sciences, University of New Mexico, Albuquerque, New Mexico 87109

[2] Department of Geoscience, University of Nevada, Las Vegas, Las Vegas, Nevada 89154

*Correspondence and requests for materials should be addressed to VJP (polyak@unm.edu).

SUPPORTING INFORMATION

S1. Hidden Cave environment

Hidden Cave, at 2000 meters elevation, is situated 600 meters higher in the Guadalupe Mountains than Carlsbad Cavern in a pinyon/ponderosa pine forest. The difference in elevation and vegetation is expressed in the calcite, where caves at higher elevation host stalagmite calcite growth having yellowish orange color, while caves a lower elevation exhibit white calcite growth (Fig. S1). Other than the color, there is no significant difference in these columnar stalagmites such as crystal fabric that is due to elevation. This difference in color is probably a reflection of the soil organic content above the caves at higher elevation having a thicker and richer soil. A description of Hidden Cave that includes tabulated temperature and relative humidity data is offered in Cokendolpher and Polyak (2004)[1]. The cave is spacious but small compared to Carlsbad Cavern. In the cave only 20-50 meters away from the entrance the temperature and relative humidity varied from 10-12° C and 87 to 95%, respectively when checked four times, one time for each season, during 1992.

S2. Stalagmite HC-1

Stalagmite HC-1 was collected in 2006 as already detached by previous visitors. Mud and charcoal in the calcite at the base of the stalagmite indicate that it likely grew on the mud floor and that the sample represents the entire stalagmite. Figure S2 shows the cross-section of the sample. Nine thin sections along the longitudinal axis exhibit a high-resolution characterization of growth where dark layers of aragonite (in transmitted light) are distinct and from which the gray value time-series was measured.

S3. Aragonite as an indicator of dry climate in stalagmite HC-1

Stalagmite HC-1 consists almost entirely of calcite growth, but exhibits numerous aragonite layers and growth hiatuses near the stalagmite top. Polyak and Asmerom (2001)[2] noted aragonite that formed on a tilted billet near a drip site in Hidden Cave (Fig. S3). Where water directly dripped onto billets lying flat under drips, calcite formed; however, on the tilted billet, aragonite formed. This demonstrated that the very thin water films on the tilted billet were prone to evaporative conditions in Hidden Cave. Aragonite layers are commonly observed on the sides of stalagmite HC-1 and other samples from this cave. In addition, a drip study in Carlsbad Cavern showed that Mg concentration in drip water increased during winter months when drip rates slowed down[3]. This suggests that the drier conditions during winter produced drip water with higher cation concentrations. The Mg/Ca ratio and CO_3 concentration in the drip water determine the mole% $MgCO_3$ in the cave calcite[4]. Hidden Cave speleothem calcite has 2.3 to 4.0 mole% $MgCO_3$[5], indicating that drip water in this cave contains abundant Mg, derived from the dolostones in which the cave formed. Lippmann (1973)[6] showed that Mg inhibits nucleation of calcite, which can promote precipitation of aragonite. Hidden Cave stalagmites contain both calcite and aragonite laminae, showing that drip water in this cave has suitably high enough concentrations of Mg to promote precipitation of aragonite, a result that was experimentally confirmed[7]. The tilted billet on which aragonite formed represented an environment where changes to thinner water films were more susceptible to the effects of evaporation that lead to higher Mg concentrations and aragonite rather than calcite precipitation. This is also illustrated on the sides of stalagmites where aragonite tends to precipitate. Accordingly, climatic changes that lead to slightly lower relative humidity in the cave and to slower drip rates can result in deposition of aragonite rather than calcite at the tops of actively growing stalagmites. Prior calcite/aragonite precipitation as stalactite growth would also be expected during periods of lower relative humidity and slower drip rates[8]. These more evaporative cave conditions that promote aragonite precipitation over calcite in Hidden Cave are interpreted to be driven by drier surface climate.

S4. Correction of aragonite $\delta^{13}C$ and $\delta^{18}O$ values for fractionation differences

We did not correct the aragonite stable isotope values for fractionation that were used in the time-series of Figure 3 and Figure S5, because we are not reporting quantitative results from our stable isotope values. Sletten et al. (2013)[9] used a routine that corrected aragonite $\delta^{13}C$ and $\delta^{18}O$ values where the measured $\delta^{13}C$ and $\delta^{18}O$ of 100% aragonite needed to be corrected by subtracting 1.7 ‰ and 0.8

‰, respectively, from the measured values. Lachniet (2014)[10] show from the fractionation equations of Kim et al. (2007)[11] that the $\delta^{18}O$ value correction is 0.38 rather than 0.8‰. In Table S2, we provide a time-series of % aragonite for which the aragonite $\delta^{13}C$ and $\delta^{18}O$ values can be corrected using the 1.7 and 0.38 ‰ corrections relative to a linear equation that incorporates the % aragonite. We show that these differences are not substantial in Figure 4A, B.

S5. Climate versus culture

The cultural history of our study area back to 4000 yr B2K using the Pecos classification is best represented by the Late Archaic, Basketmaker, and Pueblo cultures[12]. The Late Archaic and early Basketmaker intervals in the study area are also referred to as the Late Archaic/Early Agricultural period[13,14]. During the Late Archaic to Pueblo transition, societies changed in the SW USA from mostly hunter/gatherers (Archaic) to basket makers that implemented agriculture and pit houses (Basketmaker) to remarkable communities of rock building dwellers who utilized ceramics (Pueblo)[15]. The Basketmaker and Pueblo cultures are well-defined chronologically as late desert Archaic and Basketmaker II (3200 to 1500 yr B2K)[13], Basketmaker III (1500 to 1300 yr B2K), Pueblo I (1300 to 1110 yr B2K), Pueblo II (1110 to 855 yr B2K), Pueblo III (855 to 715 yr B2K), and Pueblo IV (715 to 460 yr B2K)[16-18]. The definition of Pueblo IV[16] differs up to 200 years in its length (ends at 600 yr B2K rather than 460 or 400 yr B2K) compared to those defined in the earlier literature, and other pre-Hispanic Pueblo period boundaries differ slightly depending on the source. To the east of our study area on the southern high plains, archaic cultures lacked materials to build structures that survived weathering, and therefore age models tied to these cultural traditions are less distinctly interpreted and largely rely on identification of pottery shards, projectile points, and other small lithic artifacts. In the greater Southwest, pueblo structures and pottery types are distinct, fascinating, and well-characterized.

S6. $\delta^{13}C$ and $\delta^{18}O$ time-series correlations with other Northern Hemisphere records

Stalagmite HC-1 exhibits only two distinct brief aragonite-defined droughts during the 3300 to 1040 yr B2k pluvial period, an interval that can be compared to other similar high-resolution records[19-21]. The Late Holocene in the western United States is thought to have been cooler and wetter compared to the Middle and Early Holocene[21]. Locally we have reported this to be the case[2]. Precipitation in our region is dominated by summer moisture with a strong influence by the North Atlantic Subtropical High

(NASH). In the modern, overall, warm North Atlantic temperatures during the positive phase of the Atlantic Meridional Oscillation (AMO) are associated with droughts[22]. This relationship seems to have been true during our study period. Fig. S5A shows a reasonable comparison of the HC-1 record with a temperature proxy for the North Atlantic with the speleothem $\delta^{13}C$ time-series from northern Spain[20]. This comparison, a 10-year moving average of each record from 1000 to 3300 years B2K, shows a modest but significant positive correlation ($R = 0.32$, $p < 0.05$) between HC-1 $\delta^{18}O$ and temperature, where lower $\delta^{18}O$ (wetter) values correspond to cooler temperatures. The NASH similarly strongly influences summer tropical storms in the Caribbean region, the same flow that impacts summer moisture in our region. The NASH is an ascending branch of the Hadley Cell. Warm conditions tend to weaken and shift the Hadley Cell poleward[23]. The positive correlation ($R = 0.35$, $p < 0.0001$) between the MCO1 record from Belize[19] and our record (Fig. S5B) and the temperature record from Spain (above) point to the fact that the HC-1 record reflects regional climate variability.

Supplementary References

1 Cokendolpher, J. C. & Polyak, V. J. Macroscopic invertebrates of Hidden and Hidden Chimney caves, Eddy County, New Mexico. *Texas Memorial Museum, Speleological Monographs* **6**, 175-198 (2004).

2 Polyak, V. J. & Asmerom, Y. Late Holocene climate and cultural changes in the southwestern United States. *Science* **294**, 148-151 (2001).

3 Polyak, V. J., Rasmussen, J. B. & Asmerom, Y. Drip water measurements from Carlsbad Cavern: implications towards paleoclimate records yielded from evaporative-zone stalagmites. *International Journal of Speleology* **47**, 9 (2018).

4 Gonzalez, L. A. & Lohmann, K. C. in *Paleokarst* 81-101 (Springer, 1988).

5 Polyak, V. J. *The mineralogy, petrography and diagenesis of carbonate speleothems from caves in the Guadalupe Mountains, New Mexico*, (1992).

6 Lippmann, F. in *Sedimentary Carbonate Minerals* 5-96 (Springer, 1973).

7 De Choudens-Sanchez, V. & Gonzalez, L. A. Calcite and aragonite precipitation under controlled instantaneous supersaturation: elucidating the role of CaCO3 saturation state and Mg/Ca ratio on calcium carbonate polymorphism. *Journal of Sedimentary Research* **79**, 363-376 (2009).

8 Sherwin, C. M. & Baldini, J. U. Cave air and hydrological controls on prior calcite precipitation and stalagmite growth rates: Implications for palaeoclimate reconstructions using speleothems. *Geochimica et Cosmochimica Acta* **75**, 3915-3929 (2011).

9 Sletten, H. R. *et al.* A petrographic and geochemical record of climate change over the last 4600 years from a northern Namibia stalagmite, with evidence of abruptly wetter climate at the beginning of southern Africa's Iron Age. *Palaeogeography, Palaeoclimatology, Palaeoecology* **376**, 149-162 (2013).

10 Lachniet, M. S. Are aragonite stalagmites reliable paleoclimate proxies? Tests for oxygen isotope time-series replication and equilibrium. *Bulletin* **127**, 1521-1533 (2015).

11 Kim, S.-T., O'Neil, J. R., Hillaire-Marcel, C. & Mucci, A. Oxygen isotope fractionation between synthetic aragonite and water: Influence of temperature and Mg2+ concentration. *Geochimica et Cosmochimica Acta* **71**, 4704-4715 (2007).
12 Stuart, D. & Gauthier, R. *Prehistoric New Mexico: Background for Survey*. (Historic Preservation Bureau, 1988).
13 Sliva, R. J. Cienega points and Late Archaic period chronology in the southern Southwest. *Kiva* **64**, 339-367 (1999).
14 Huckell, B. B. The archaic prehistory of the North American Southwest. *Journal of World Prehistory* **10**, 305-373 (1996).
15 Fiedel, S. J. *Prehistory of the Americas*. (Cambridge University Press, 1999).
16 Bocinsky, R. K., Rush, J., Kintigh, K. W. & Kohler, T. A. Exploration and exploitation in the macrohistory of the pre-Hispanic Pueblo Southwest. *Science advances* **2**, e1501532 (2016).
17 McCaffery, H., Tykot, R. H., Gore, K. D. & DeBoer, B. R. Stable isotope analysis of turkey (Meleagris gallopavo) diet from Pueblo II and Pueblo III sites, middle San Juan region, northwest New Mexico. *American Antiquity*, 337-352 (2014).
18 Adler, M. A. *The Prehistoric Pueblo World, AD 1150-1350*. (University of Arizona Press, 2016).
19 Akers, P. D. *et al.* An extended and higher-resolution record of climate and land use from stalagmite MC01 from Macal Chasm, Belize, revealing connections between major dry events, overall climate variability, and Maya sociopolitical changes. *Palaeogeography, Palaeoclimatology, Palaeoecology* **459**, 268-288 (2016).
20 Martín-Chivelet, J., Muñoz-García, M. B., Edwards, R. L., Turrero, M. J. & Ortega, A. I. Land surface temperature changes in Northern Iberia since 4000 yr BP, based on δ13C of speleothems. *Global and Planetary Change* **77**, 1-12 (2011).
21 Lachniet, M. S., Asmerom, Y., Polyak, V. & Denniston, R. Great Basin Paleoclimate and Aridity linked to Arctic warming and tropical Pacific sea surface temperatures. *Paleoceanography and Paleoclimatology*, e2019PA003785 (2020).
22 McCabe, G. J., Palecki, M. A. & Betancourt, J. L. Pacific and Atlantic Ocean influences on multidecadal drought frequency in the United States. *Proceedings of the National Academy of Sciences* **101**, 4136-4141 (2004).
23 Lu, J., Vecchi, G. A. & Reichler, T. Expansion of the Hadley cell under global warming. *Geophysical Research Letters* **34** (2007).

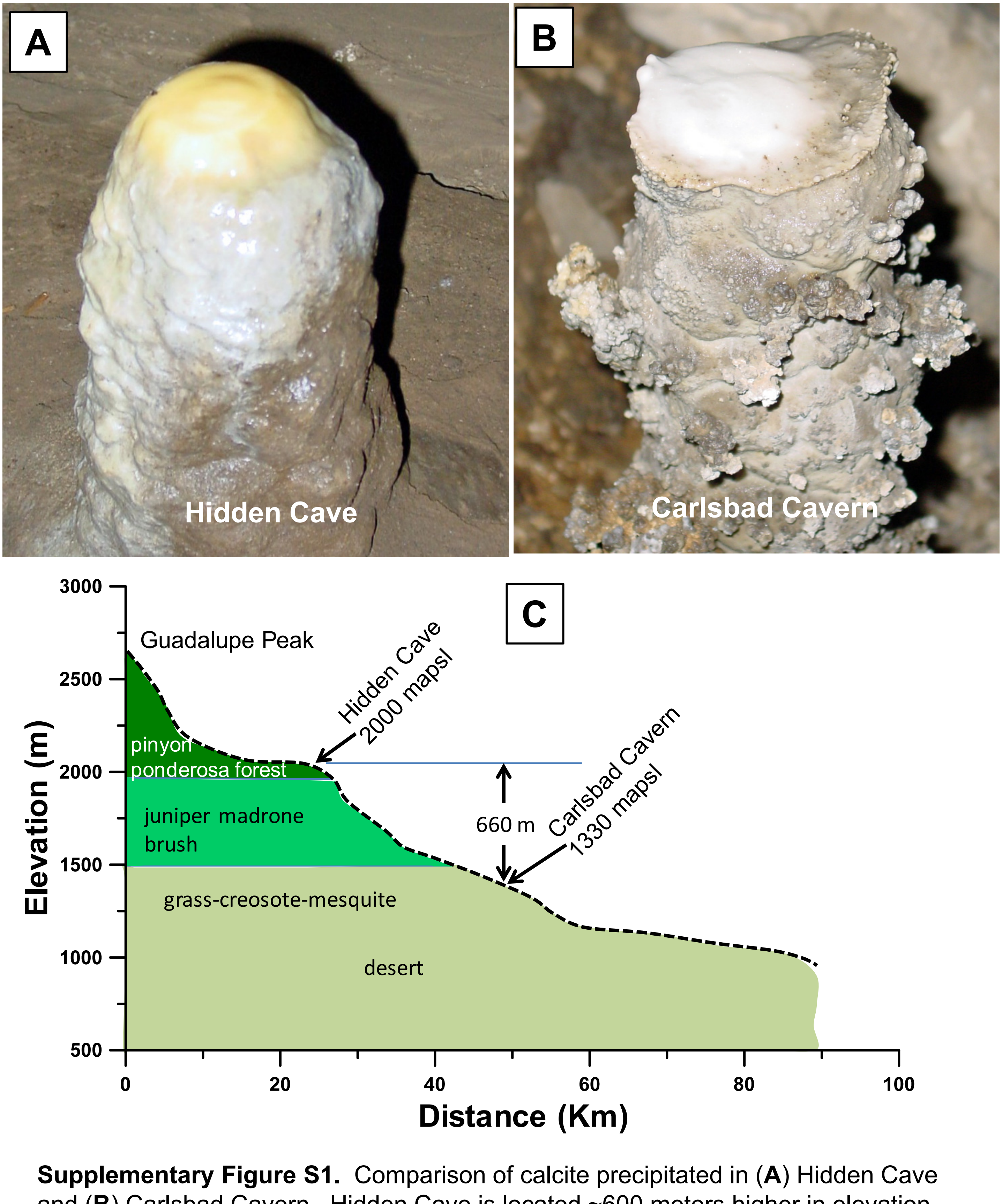

Supplementary Figure S1. Comparison of calcite precipitated in (**A**) Hidden Cave and (**B**) Carlsbad Cavern. Hidden Cave is located ~600 meters higher in elevation than Carlsbad Cavern, in a pinyon pine forest that receives roughly 100 mm more rain and snow annually. (**C**) The Carlsbad Cavern area is not forested. The yellowish orange calcite in Hidden Cave stalagmites reflects this difference in elevation and vegetation. mapsl = meters above present sea level. Images by V.J. Polyak.

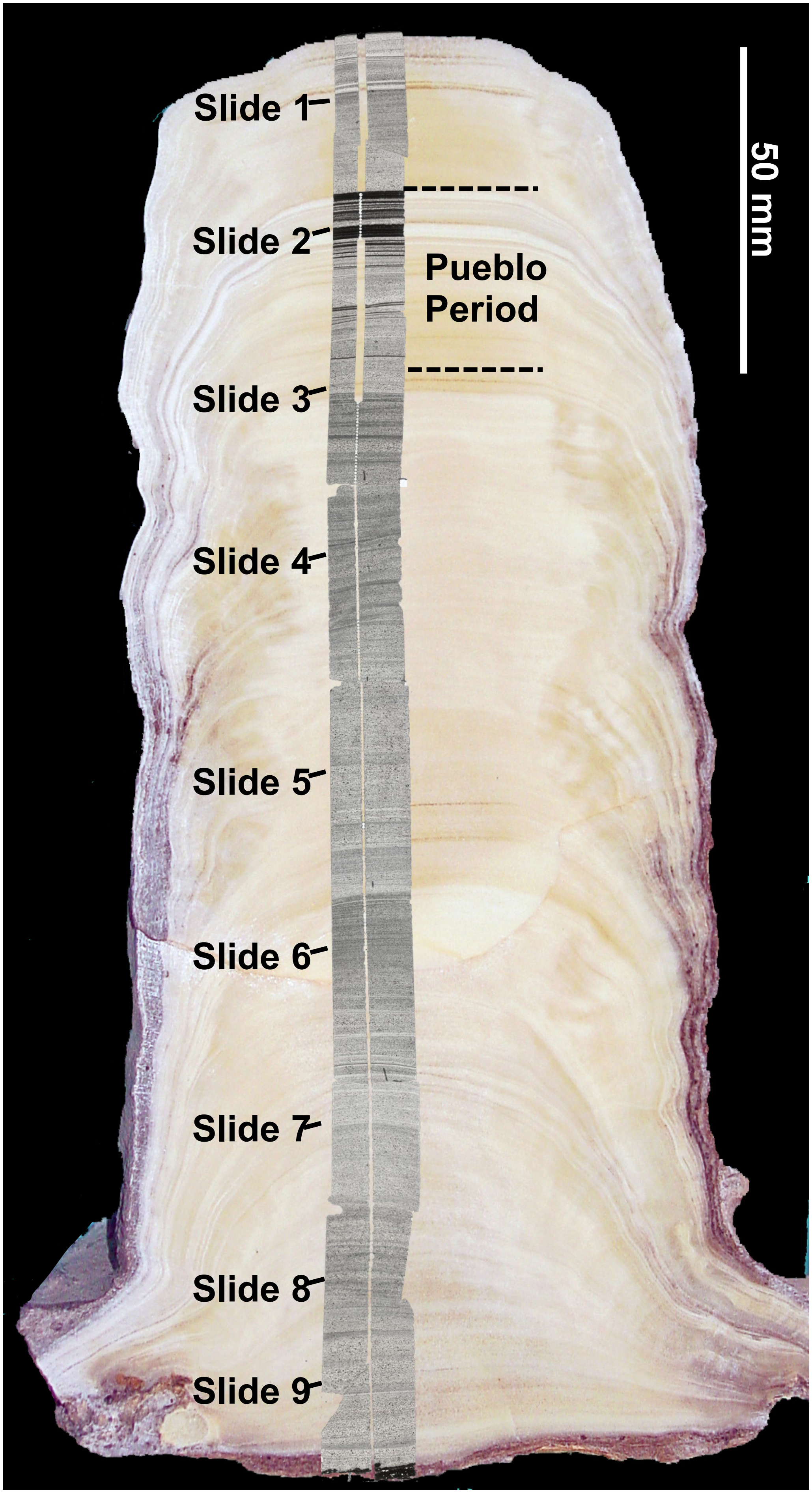

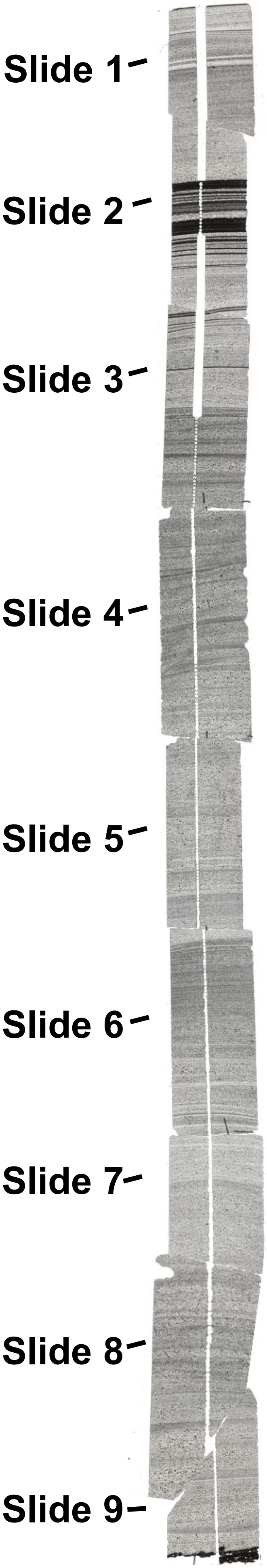

Supplementary Figure S2. Stalagmite HC-1 overlain with nine thin sections. Note the stable isotope transect in the center of the thin sections. The stalagmite is 0.225 m in length. Images by V.J. Polyak.

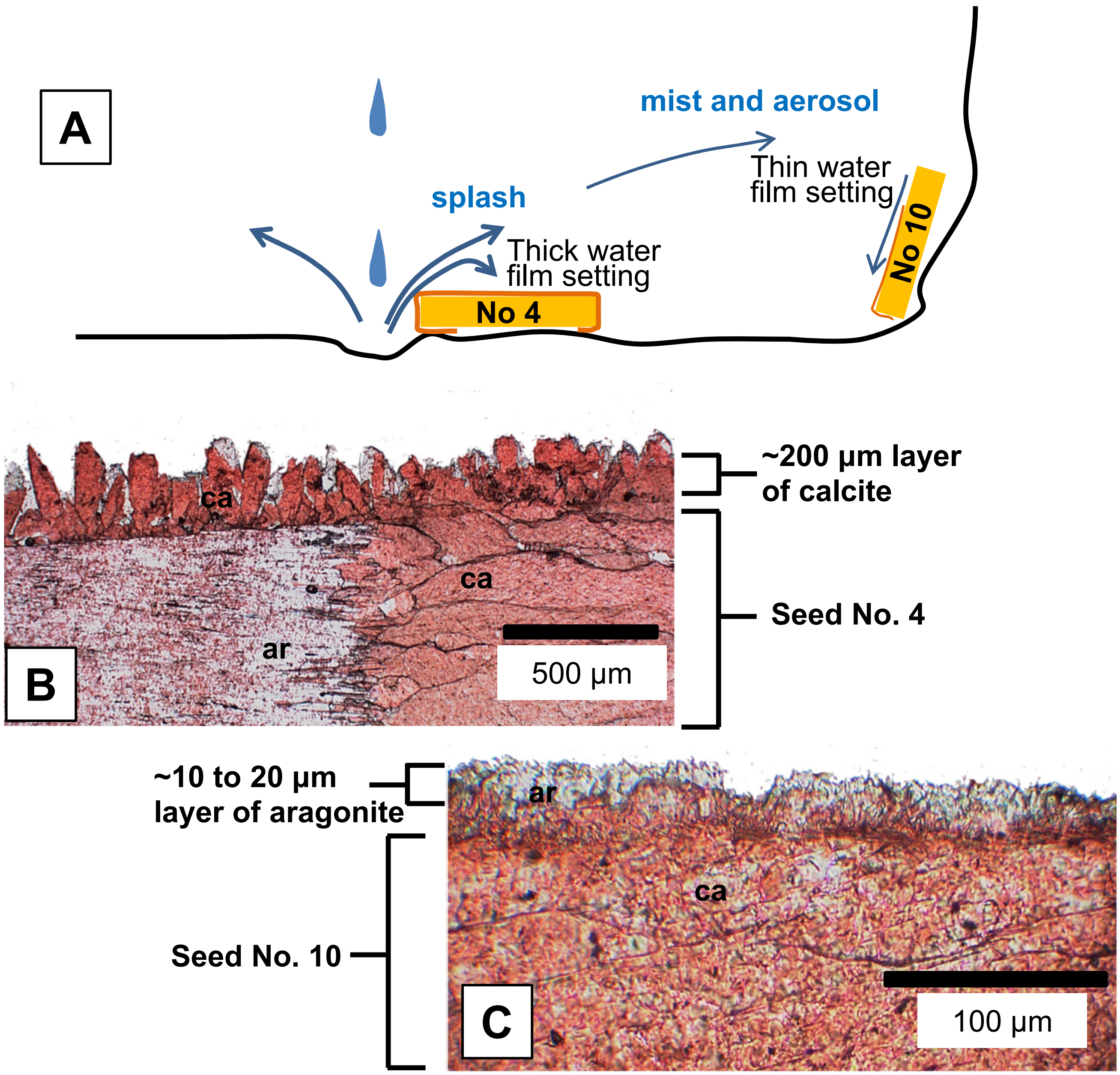

Supplementary Figure S3: Two billets (No. 4 and No. 10) were placed in Hidden Cave as seeds to observe calcite growth from cave drips. **(A)** The cave setting for the seeds is a drip site, where seed no. 4 was placed flat on the cave floor very close to the drip site, while seed No. 10 was placed upright much further from a drip site. Seed No. 4 receiving splashing water, while seed No. 10 received mist and aerosol from the dripping water. **(B)** Thin section image showing calcite that formed on seed No. 4. Note that calcite formed on both aragonite (ar) and calcite (ca). The layer of calcite on seed No. 4 over a year's span was ~200 µm thick and formed from a thick water film fed by splashing water. **(C)** Thin section image of seed No. 10 showing a thin layer of aragonite that formed on the calcite seed over a year's span. Seed No. 10 was fed by mist and aerosol creating a thin water film that was undoubtedly more influenced by evaporation. ca = calcite and ar = aragonite. Images by V.J. Polyak.

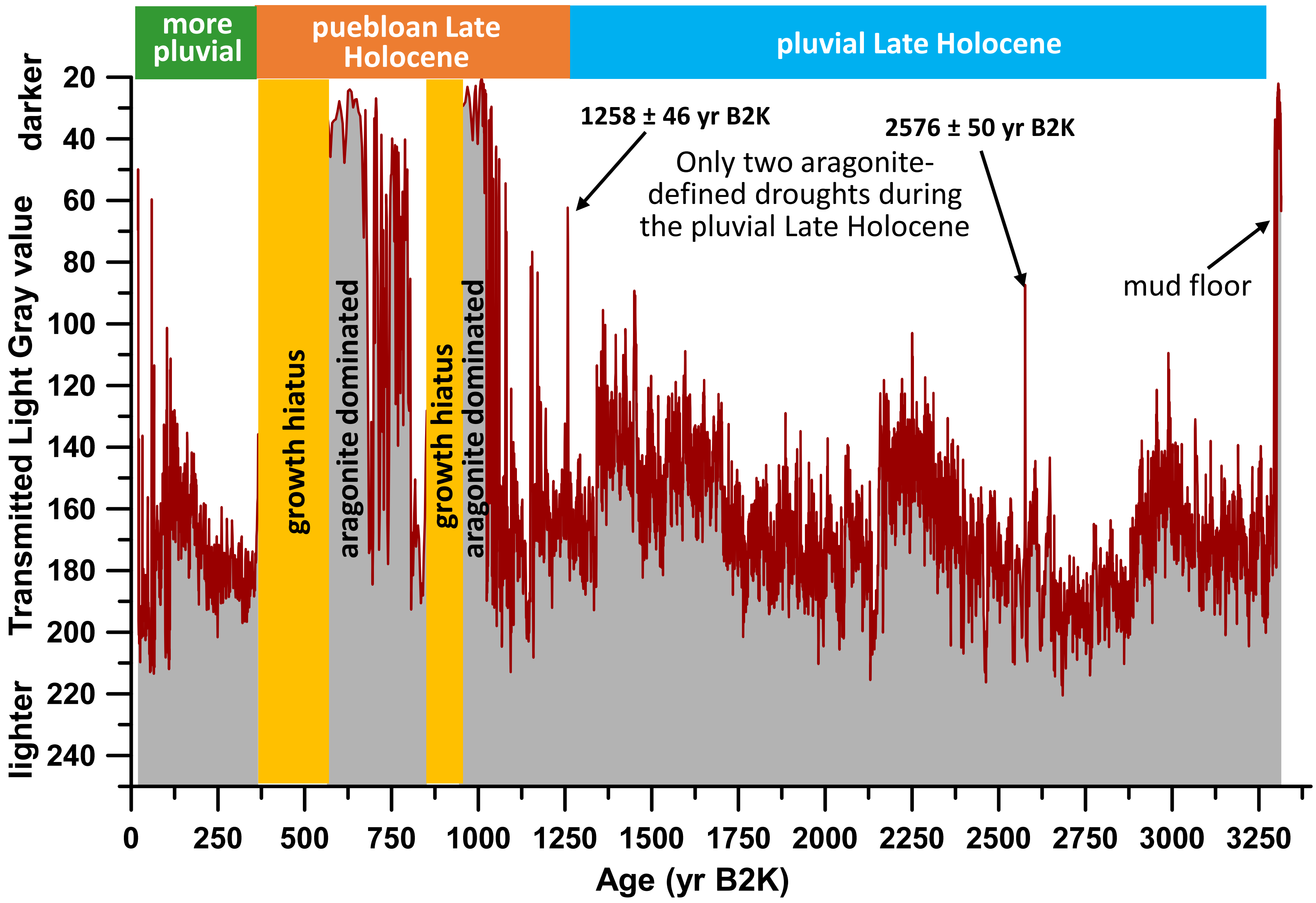

Supplementary Figure S4. Stalagmite HC-1 grayscale time-series shows the character of growth that is used to suggest three sub-periods, pluvial, puebloan, and return to pluvial, of the Late Holocene period. From the beginning of stalagmite HC-1 growth up to 1260 yr B2K, only two aragonite-defined droughts occur during pluvial Late Holocene sub-period.

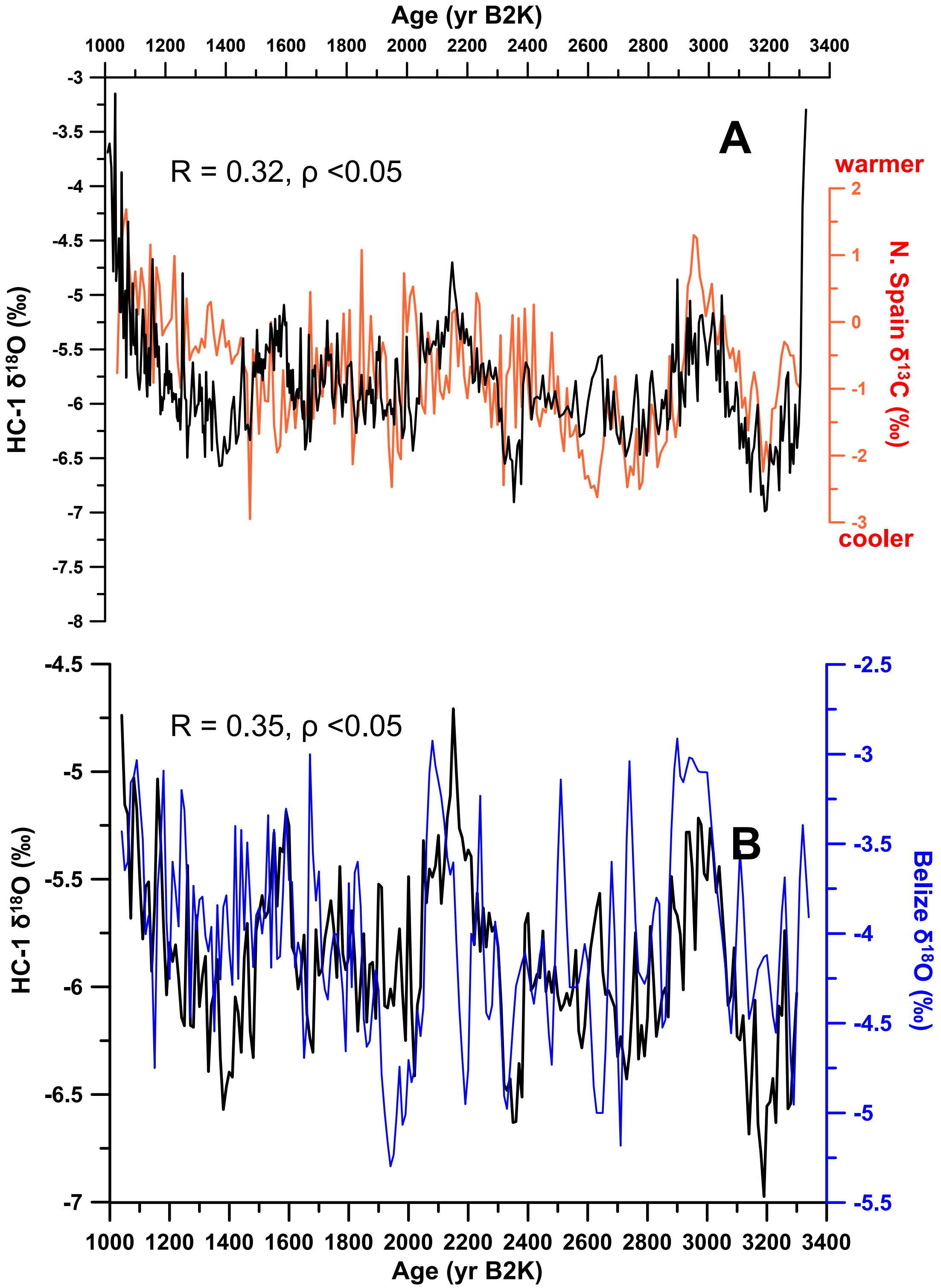

Supplementary Figure S5. Comparison of $\delta^{18}O$ and $\delta^{13}C$ isotope time-series to those of two other northern hemisphere records[19,20] that show modest but significant correlations at 10-year moving averages. We propose that the importance of the correlations are that the stalagmites are recording a teleconnection of paleoclimate occurring in different regions of the hemisphere related to northern hemisphere temperature and other climate oscillations. See section **S6**.

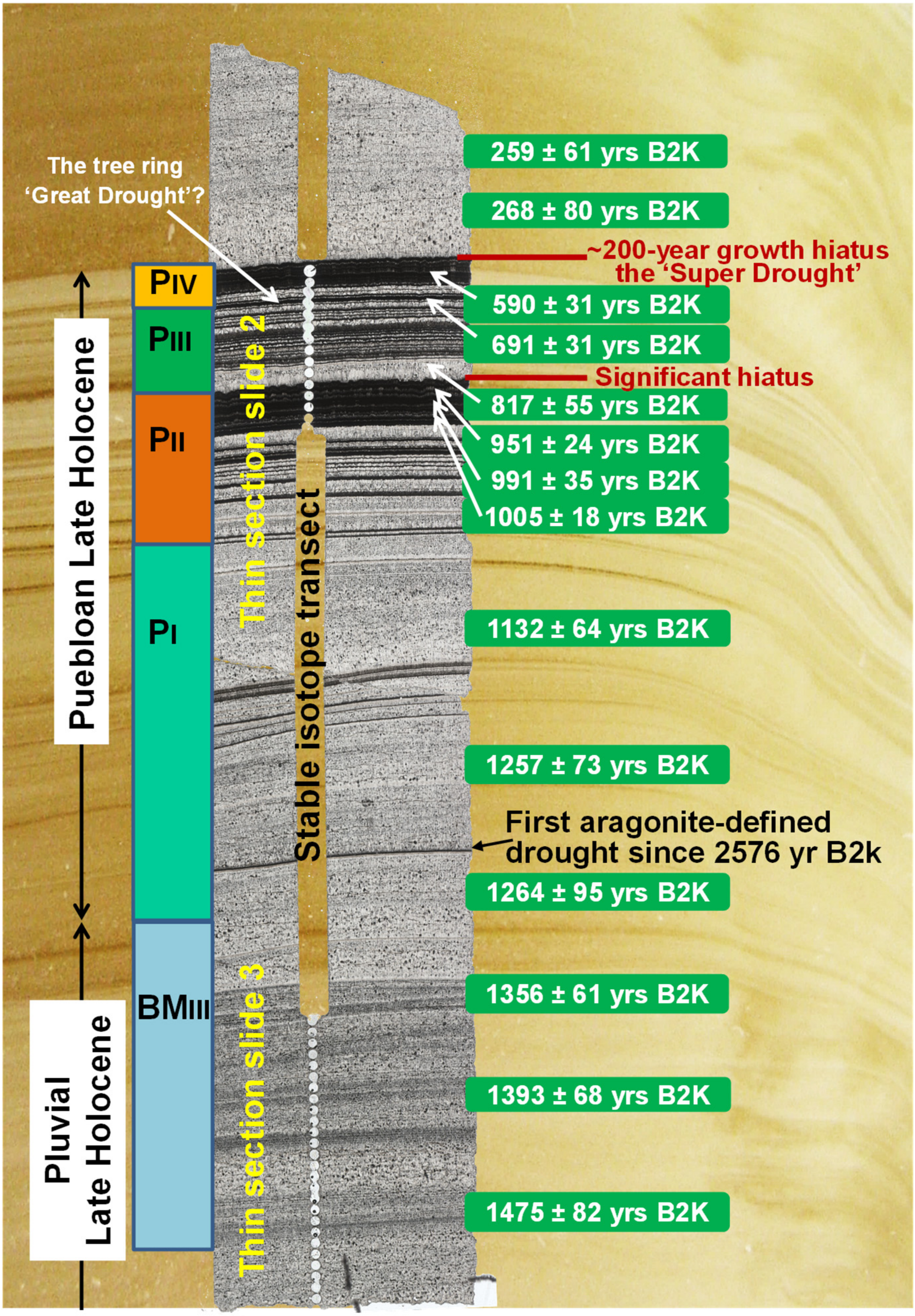

Supplementary Figure S6. Transmitted-light images of the top two thin sections placed on a reflected light image of stalagmite HC-1 show the sharp contrast in growth of the stalagmite through the Pueblo period. The two thick aragonite layers each terminating in a thick growth hiatus are coeval with Pueblo II and IV, which are interpreted as the driest intervals of the pueblo Late Holocene. The Pueblo period is coeval with all aragonite layers/growth hiatuses in stalagmite HC-1 and considered as the driest climatic sub-period of the last 3400 years of the Late Holocene. Images by V.J. Polyak.
